# Narrowing the Definition of Social Inclusion in Sport for People with Disabilities through a Scoping Review

**DOI:** 10.3390/healthcare11162292

**Published:** 2023-08-14

**Authors:** Viktorija Pečnikar Oblak, Maria João Campos, Susana Lemos, Micaela Rocha, Predrag Ljubotina, Kaja Poteko, Orsolya Kárpáti, Judit Farkas, Szilvia Perényi, Urška Kustura, Alain Massart, Mojca Doupona

**Affiliations:** 1 Faculty of Sport, University of Ljubljana, 1000 Ljubljana, Slovenia; viktorija.pecnikar-oblak@fsp.uni-lj.si (V.P.O.); predrag.ljubotina@fuds.si (P.L.); kaja.poteko@fsp.uni-lj.si (K.P.); mojca.doupona@fsp.uni-lj.si (M.D.); 2 Faculty of Sport Sciences and Physical Education, University of Coimbra, 3040-248 Coimbra, Portugal; 3 Research Center for Sport and Physical Activity (CIDAF), 3040-248 Coimbra, Portugal; 4 Portuguese Association for Developmental Disorders and Autism of Coimbra (APPDA Coimbra), Hospital Pediátrico de Coimbra, Av. Afonso Romão, 3000-602 Coimbra, Portugal; susana.lemos.appdac@gmail.com (S.L.); micaela.rocha.appdac@gmail.com (M.R.); 5 School of Advanced Social Studies Nova Gorica, Gregorčičeva Ulica 19, 5000 Nova Gorica, Slovenia; 6 Special Olympics Hungary, H-1146 Budapest, Hungary; karpati.orsolya@msosz.hu; 7 Quality Assurance and Accreditation Office, Hungarian University of Sports Science, H-1123 Budapest, Hungary; farkas.judit@tf.hu (J.F.); perenyi.szilvia@tf.hu (S.P.); 8 Special Olympics Slovenia, Samova 9, 1000 Ljubljana, Slovenia; urska.andrejc@gmail.com

**Keywords:** athletes, disability, vulnerable people, policy, practice

## Abstract

The concept and practice of social inclusion in sport are still undefined, causing confusion both in the field of sport policy and practice. According to the United Nations (UN), a conceptual and analytical work on what constitutes inclusion is needed. Therefore, this study aims to define social inclusion in sport for people with disabilities by reviewing the existing literature. Using a scoping review framework, articles related to a possible definition of social inclusion in sport or to the elements of this definition were reviewed. For the eighteen (18) articles selected, the focus was on 152 statements, which were grouped into 6 main categories, namely: policy (29), fundamental conditions (28), key elements (30), soft skills (20), field gaps (31), and best practices (14). Ten keywords were extracted from each of the six categories using the free online program cortical.io. All 60 keywords were then compared with each other. After deleting the duplicates, 24 keywords remained, which were classified into five major categories: (1) key people, (2) key environments, (3) key ways to use, (4) key benefits, and (5) key barriers, in order to create a descriptive definition of social inclusion in sport for people with disabilities that can contribute to the goals of the UN 2030 Agenda. In addition to the definition, relevant issues were also raised for in-depth discussion and further research.

## 1. Introduction

Sport is an effective tool for the social inclusion of people with disabilities, and the United Nations (UN) has recognized sport in its 2030 Agenda [1] as an important contributor to the realization of sustainable development and peace goals due to its promotion of tolerance and respect and facilitation of social inclusion, conflict prevention, and peacebuilding. For the purposes of this paper, “sport” refers to all forms of physical activity that, through occasional or organized participation, aim to express or improve physical fitness and mental well-being, forming social relationships or obtaining results in competition at all levels [2]. The UN Convention on the Rights of Persons with Disabilities recognizes “that disability is an evolving concept and that disability results from the interaction between persons with impairments and attitudinal and environmental barriers that hinders their full and effective participation in society on an equal basis with others” [3].

Social inclusion enables all members of the community to acquire vital skills, develop a sense of belonging, and gain independence [4]. It is a process of improving the conditions for participation in society, especially for people who are disadvantaged [5], by improving opportunities, access to resources, voice, and respect for rights. While inclusion is a central goal of the 2030 Agenda, conceptual and analytical work on what constitutes inclusion is needed, as well as efforts to improve data availability [6]. Thus, governments, policy makers, and community leaders should engage other stakeholders, such as private companies, non-governmental organizations, new social movements, and campaign groups, to improve social inclusion, especially for people with disabilities [4,7,8,9,10,11,12,13]. In the context of sport, the concept of social inclusion embraces the heterogeneity of athletes with disabilities and takes their diversity as a starting point for inclusive sport theory and practice. Consequently, the concept is defined and measured in different ways [14].

There are a growing number of global calls for action for promoting physical activity and sport among people with disabilities (e.g., [1,15,16,17,18]). The UN recognizes that people with disabilities have a fundamental right to “full and effective participation” in society, including in sport. The Convention on the Rights of Persons with Disabilities highlights, in Article 30, the right of persons with disabilities to participate on an equal basis with others in cultural life, including recreational, leisure, and sporting activities, and to have the opportunity to access and participate in general sports activities at all levels, as well as disability-specific sports and leisure activities [3].

However, there is a gap between policy and practice. The lack of clear wording in policy and the use of vague terminology lead to a misconception of how to operationalize inclusion in practice and generates space for environmental and social barriers that limit the participation of people with disabilities in sport and increase their marginalization and discrimination in society [8,11,13,19,20,21,22,23,24].

Although there is no clear definition of sport and inclusion [10,20] for people with disabilities, it can no longer be said that there is a lack of academic interest in the field of social inclusion in sport [25,26,27]. For example, using keywords in Google Scholar such as disability or impairment, children and youth, sports clubs, sport, or organized sport and inclusion, approximately 13,100 reviewed articles were found in the databases over the last ten years [28]. Our review found a wide variety of methods for studying the topic of sport inclusion of people with disabilities, using questionnaires [12,24,29] and/or structured, semi-structured interviews or narrative inquiry [11,24,25,30], Moreover, a confirmatory questionnaire was created to assess the involvement in sport, with an analysis based on theoretical foundations such as the social model of disability, the definition of abilities, the nature of social inclusion/exclusion, sources of motivation, the form of social support, the theory of planned behavior, DeLuca’s four conceptions of inclusion [31], the block model of empowerment, and social field theory [8,11,13,24,28,29].

The research also identifies the perceptions of parents, coaches, disabled people, athletes with disabilities, other partners in sport, management of voluntary clubs, sports organizations responsible for policy, and other groups at risk of social exclusion in European and global countries such as Serbia, Poland, Ukraine, Germany, Hungary, the United Kingdom, Greece, Italy, Belgium, Sweden, Denmark, England, the Netherlands, Portugal, Norway, Spain, Switzerland, Australia, Africa, the USA, and Asia [8,11,13,24,25,28,29,30,31,32].

There is a need to seek the existing scientific literature for elements of reflection that may contribute to a better definition of the social inclusion in sport for people with disabilities. Despite the great interest of policy makers and academics in the inclusion of people with disabilities, there are no universally accepted definitions of inclusion in the sport literature. This gap contributes to the imprecision of policies [13,23], providing several stakeholders, such as national federations, mainstream sports clubs, among others, a wide degree of freedom in interpreting what constitutes inclusion in their context. Our aim is therefore to analyze the existing literature through a scoping review and propose elements for a definition of social inclusion in sport for people with disabilities so that stakeholders could have a consistent approach to social inclusion in sport and thus support all athletes to reach their full potential, regardless of their abilities.

## 2. Materials and Methods

### 2.1. Protocol and Search Method

This review was conducted using scoping review methods, as scoping reviews are often conducted to examine and clarify definitions used in the literature [33,34]. In addition, the identification and analysis of knowledge gaps is a common and valuable indication for conducting a scoping review [34]. Four review components were used (1) identification of the research question; (2) identification of relevant search records; (3) record selection for inclusion; (4) extraction and report of the results.

### 2.2. Identification of the Research Question

Using the Population, Intervention, Comparator, Outcome Study (PICOS) design [35,36], we arrived at the research question: What elements can be used for a definition to promote the social inclusion of people with disabilities in sport?

### 2.3. Identification of Relevant Search Records

**Figure 1:**
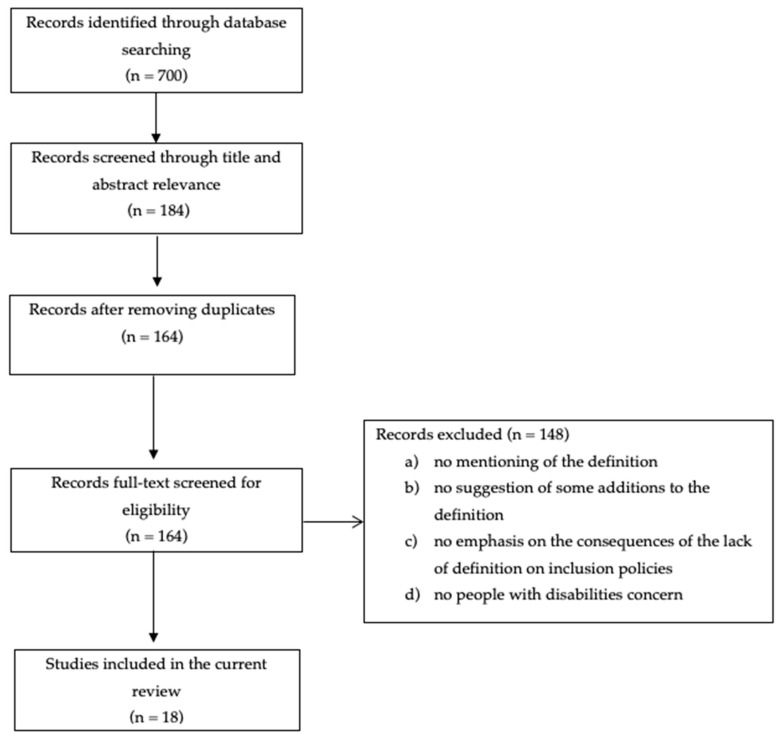
PRISMA flow chart for the scoping review process [37].

To identify evidence, a preliminary literature search was carried out in the Google Scholar search engine to take advantage of the versatility of the sources. Several keywords and strings were tested, and it was found that there is a difference between the two terms “inclusion in sport” and “social inclusion in sport”. Depending on the scope of the paper, articles dealing with social inclusion seemed to be more appropriate. Even with the search string definition of social inclusion in sport in the title, we came across non-existent articles, so we tried to replace the word definition with another, such as “define” or “normative”. In the end, the set “social inclusion and sport or mainstream sport and disability and finance or policies or definitions” was used in the period between 2012 and 2022, generated 2370 articles. The results were sorted by the appropriateness of the titles by three members of the research team with expertise in adapted physical activity. The team selected 200 links and after analysis, 150 titles were rejected. After reviewing the 50 selected articles, another 100 interesting articles emerged from the references. Finally, we came up with 88 articles, but only a few of them were relevant to our research question. Based on this preliminary literature search, we conducted a structured search for relevant studies in four scientific databases (B-on, Google Scholar, PubMed, and Scopus) for the period up to 20 July 2023, using the following search keywords and terms: “social inclusion in sport” or “social inclusion in sport” and “policy” or “policies” and “disability” or “disable” (social inclusion in sport* and polic* and disab*). From 700 links opened, based on titles and abstracts, two members of the research team independently selected eligible articles in the databases, and after removing 20 duplicates, reached 125 in Google Scholar, 22 in B-on, 10 in PubMed, and 7 from Scopus (Figure 1).

### 2.4. Records Selection for Inclusion

The 164 eligible articles were screened for inclusion by two members of the research team who read the full texts, yielding eighteen (18) full text articles included (Figure 1). After comparing the included articles, in case of doubt, three other members of the team were consulted to make the final decision.

The inclusion criteria used were: people with disability, as a population; social inclusion in sport (mainstream, unified sport, community sport) as context; “tentative of” or “elements for” a definition of social inclusion or policies in sport, for people with disabilities (PWD) or with the potential to help PWD, as a core concept of the scoping review; and publication in peer review periodic (observational and experimental studies, reviews, meta-analyses) in English or the language of the research team as the type of sources.

### 2.5. Extraction and Report of the Results

For all the selected articles, in a first table, were collected the first author and the year of publication in the first column, the title of the article in the second column, the elements important for defining the research topic in the third column. This was followed by a process of reviewing and classifying the funded elements for a definition, into meaningful, similar categories. Six categories were identified by members of the research team, all of whom have experience of adapted physical activity. A second table was created with the six categories in the first column, the key statements of each category in the second column, and 10 extracted definition keywords by category in the third column [38]. The 10 extracted definition keywords were obtained using the free Extract Keywords program from cortical.io (https://www.cortical.io/freetools/extract-keywords/ (accessed on 26 July 2023)). Then, all 60 keywords were compared, and the duplicates were deleted, leaving 24 words, all of which were used to define social inclusion in sport. In a third table, these 24 remaining words were clustered by the research group into 5 keyword groups to facilitate the construction of a successful definition, depending on who the crucial people are, where the best environment is, how best to use it, what the benefits for people are, and what main barriers to avoid.

## 3. Results

In an attempt to define social inclusion in sport for people with disabilities based on keywords, 152 important statements were extracted from the selected articles for creating a definition. Table 1 shows only the first three statements from each study. The full statements in each article selected by the research team can be found in the Supplementary Material (Table S1).

The 152 statements reached were processed into six key categories, namely: policy (29), fundamental conditions (28), key elements (30), soft skills (20), field gaps (31), and best practices (14). Ten keywords were extracted from each of the six categories using the free online program cortical.io. Table 2 shows only a subjective view of the interesting statements selected by the research team. The full statements included in each category can be found in the Supplementary Material (Table S2). The ten extracted definition keywords are listed in Table 2.

Next, all 60 keywords were compared, and after deleting the duplicates, 24 keywords remained, which were reclassified into five important categories: (1) key people, (2) key environments, (3) key ways to use, (4) key benefits, and (5) key barriers (Table 3).

Consequently, the following descriptive definition of social inclusion in sport for people with disabilities was defined: “Social inclusion in sport is a key approach that ensures all individuals, regardless of their abilities/disabilities or background, can actively participate in sporting activities within mainstream sports organizations. Coaches cater to individual needs, promoting mixed-ability activities to provide equal opportunities for athletes and other participants, like volunteers. The benefits include enriched experiences, improved skills, and a sense of belonging. Researchers’ findings and coaching outcomes support this approach, revealing its positive impact on participants. However, obstacles such as exclusionary norms, attitudinal barriers, and inadequate policies may hinder full participation”.

## 4. Discussion

In the absence of a clear definition [46], attempting to define the concept of social inclusion of people with disabilities in sport will help policy makers implement more effective inclusive sport programs. Eighteen (18) studies were selected that included or suggested a definition. To try to establish a definition, we collected 24 of the most commonly used related keywords in the scientific literature.

### 4.1. The Attempt to Define Social Inclusion in Mainstream Sport for People with Disabilities

Based on the keywords, a definition of social inclusion of people with disabilities in sport was developed:

*“* *Social inclusion in sport for people with disabilities is a key approach that ensures all individuals, regardless of their abilities/disabilities or backgrounds, can actively participate in sporting activities within mainstream sports organizations. Coaches cater to individual needs, promoting mixed-ability activities to provide equal opportunities for athletes and other participants like volunteers. The benefits include enriched experiences, improved skills, and a sense of belonging. Researchers’ findings and coaching outcomes support this approach, revealing its positive impact on participants. However, obstacles such as exclusionary norms, attitudinal barriers, and inadequate policies may hinder full participation.”*

We are aware that some essential aspects for the success of social inclusion in sport are not included in this definition, which highlights that studies may not focus enough on what can really contribute to the effective social inclusion of people with disabilities in sport.

The attempted definition does not say much that is new, nor does it highlight any aspects that are not already known. Instead, it frames and narrows the field. It certainly lacks more specific instructions, such as who is responsible or, as Dyer & Standford emphasize, that sport should be regular, frequent, and sustained [52]. Our paper is more a collection of researched areas such as policies, basic conditions, key elements, known soft skills, field gaps, and best practices in one place. We do not process the collected material in the form of new knowledge. Instead, we focus on creating a framework definition as a starting point from which we can develop a more structured social inclusion policy in sport with a vision, strategy, model, and implementation at local and national levels.

Depending on their preferences and needs, people with disabilities’ choices to participate in sport can vary greatly: some prefer to train in mainstream clubs because there they can train with people without disability; others feel more comfortable in segregated activities, parallel activities, or mixed activities. Many scholars have argued for giving equal importance to segregated and inclusive approaches, arguing that many people who engage in disability-specific sport may regain their self-confidence, which later enables them to engage in mainstream sport, in a sport development continuum [4,45,46,55]. It is perhaps too early to claim that true inclusion in sport takes place in sports clubs involving groups with athletes of different abilities, yet there are many authors who support this idea. We can read about the importance of full and partial inclusion [46], mixed ability groups [52], the empowerment model [28], and sport for all [42,45,47].

Kiuppis [4], starting from the inclusion debate in education as the main reference context, has established some basic definitions of sport, disability, quality physical education, and physical literacy, which are the starting point for defining social inclusion in sport. The work is based on four aspects: (1) the aspect of participation, (2) a minimum standard of sport for all, (3) the links between inclusion in sport and quality physical education, disability, and participation, and (4) the consideration of different concepts of inclusion [4]. This work represents a step forward and focuses more on inclusion in afternoon recreational sport activities that generally take place in sports clubs [49]. By moving forward, the focus is more on: (1) narrowing the functional definition of social inclusion in sport, (2) highlighting the gaps in this area that lead to a lack of implementation of social inclusion in sport, and (3) understanding the evolving language to avoid misconceptions.

In order to provide stakeholders and researchers with some insights for a more efficient definition, our research team has selected essential statements from all categories that were not reflected in the 24 keywords. Based on this selection, we highlight topics of interest for further research and deeper discussion.

### 4.2. Misunderstandings and Vagueness

Especially in the last three years, research in the field of social inclusion in sport has expanded considerably, as it is a new concept for decision makers, while good practices based on volunteering have existed since the 1950s. On the other hand, people with disabilities are still considered unable to function as members of normal society in many Asian countries [56]. There is still no consensus among scholars, practitioners, and policy makers on what constitutes social inclusion [45]. The idea and concept of inclusion of para-sports is also not defined [50]. Furthermore, “normalized” and exclusionary concepts and practices in youth sport need to be critically challenged [57]. Anderson et al. [50] state that opinions have been expressed that inclusion is an unattainable, utopian goal, but they go on to state that this may be due to a lack of knowledge about accountability and implementation. The results show a discrepancy in the perception of inclusion. They go on to say that no one dares to raise their hand and ask not only what inclusion means, but also how it should be implemented and what should be preserved in the process [50]. Furthermore, according to Townsend et al. [49], the question of whether disabilities should be addressed in separate blocks or integrated into the structures of regular education is still hotly debated [49].

### 4.3. The Difference between the Mainstream Sports Environment and Specialized Institutions

There is a significant difference between inclusion in the mainstream sport environment and in specialised facilities for people with disabilities or in para-sport. Hums et al. [58] point out that it is important to note that disability sports organizations (DSOs) are organized and named by disability (cerebral palsy, hearing or visual impairment, etc.), whereas national governing bodies are named by sport. The DSOs have a somewhat better regulated policy and basis, while in sport, this part is just being established through the social inclusion policy. However, the selected articles do not talk about funding, preferring to use terms such as budgetary constraints, lack of available support staff and resources for capacity building, sharing resources and gaining political support, implementing collaborative shared leadership through the joint development and implementing action plans, and re-conceptualizing ideas about responsibility [51].

Research in a group of mixed ability athletes emphasized that the key to inclusion is a welcoming, supportive general environment, regular and sustained provision, equal membership, and the promotion of self-determination to maximize positive outcomes such as changing perceptions of disability, developing friendships, and promoting personal development [52]. Other health and social benefits were key factors in prolonged engagement in wheelchair basketball, and it was reported that reverse integration led to better mutual understanding of the impact of (dis)ability [59]. Ester et al. [53] also say that participation in sport has a positive impact on psychological well-being, indicators of psychological ill-being, and social outcomes in adults.

There is also an important shift from the traditional disability model to the social model of caring for people in the community. Sofokleous & Stylianou [60] state that social model stimuli had a positive effect on pro-disability policy attitudes, and medical model stimuli had a negative effect on pro-disability policy attitudes. Townsend et al. [49] write that in specialized or separate institutions for people with disabilities, the medical model of approach, treatment, and mindset is very present [49]. The definition of social inclusion in sport is therefore based more on the social model that supports sport for all, the empowerment model, and mixed ability sport [28,42,45,47,52].

Many articles also refer to the Unified Sport of the Special Olympics [39,42,52] as one of the best practices of social inclusion in sport. Best practices represent one of the developed categories in this article. It is interesting to note that there are few best practices compared to the number of other statements (n = 14). And precisely because Unified Sport, as an activity of a specialized institution for athletes with intellectual disabilities, is so “sung”, it is at the same time, an ideal demonstration of the mixed-ability approach that should be implemented in sports clubs, where it is still in its infancy from the perspective of mass practice.

Successful social inclusion, therefore, requires a change to the social model [46,49], and this is most likely to succeed in mainstream sport.

### 4.4. The Language and the Explanation of the Key Words Used

In defining social inclusion in sport, it was necessary to understand some terms in a new way. Even though the concept of inclusion is well established in other professional fields such as education and employment, it is new in sport [4,61].

The literature review also revealed that different professional fields such as education, employment, social security, health care, etc. think and write about inclusion somewhat differently. The meaning of inclusion also varies, depending on how it is used and understood in different countries. The “new” phrases about social inclusion in and through sport, written in English and also more characteristic of English speakers, are not necessarily understood in the same way in other languages. Even a search of the literature on inclusion in sport showed us that one has to specify the term “social inclusion” to get the right results in the browser, as the word “inclusion” itself is too broad. All this contributes to the vagueness of the field, which is also noticeable in the legal framework [46].

The language of social inclusion in sport should be very positive and chosen to support anyone interested in regular sport and exercise. Both the terminology and the approach go beyond the mere use of the body and emphasize, above all, the social touch in the sense of social action, social networks, and social capital that excludes no one [32,45].

### 4.5. Social Inclusion in Sport

Thus, social inclusion in sport is the most comprehensive concept and refers to all athletes and others involved in sport, such as parents, volunteers, coaches, managers of sports organizations, sports federations, as well as specialized entities such as organizations for the disabled, asylum seekers’ homes, humanitarian organizations, sponsors, and donors [4,28,46,47]. It includes all types of sports, from weekly recreational sports to competitive sports. Social inclusion in sport means caring for all who participate in sport, especially the vulnerable [28,40,42,46,47]. These may be children, adolescents, or adult athletes who need adjustments due to various psychophysical conditions, illnesses, or other personal circumstances. They may be in a socially vulnerable situation, face resettlement, have refugee status, belong to a different ethnic minority, and therefore have fewer opportunities, face injury, end their elite sports career and find themselves in a new situation they are not familiar with, have difficulty balancing sports and school or sports and work in the so-called dual career system and others. In the search for a definition, we have deliberately avoided dividing sport into sport and para-sport or sport for people with disabilities. The concept of social inclusion in sport is also characterized by the fact that it encompasses sport as a whole [42,43].

### 4.6. Mainstream Sport Environment

The term “mainstream sport” is deliberately chosen in the definition to refer to participation in sport in majority, dominant, traditional, and mass sport organizations, such as sports clubs, rather than specialized, segregated, minority, or disabled organizations, such as disability associations. While the latter are not excluded, they are intentionally not highlighted because they already exist and are “too entrenched”. Since segregation is so historically present, the attempted definition emphasizes the place where inclusive sports programs should take place [46]. We called it the mainstream sport environment, and it includes mainly classical sport organizations, such as sport clubs, because it is well known that para-sport in Europe is mainly practiced in special organizations with disability status [19,28,39,43,46]. But the situation in this field is already changing. The trend is for all world sports federations to take para-sport disciplines under their wing (Source: IPC members approve new constitution at General Assembly (paralympic.org)).

### 4.7. Important Persons

Using our keywords, most studies were found that refer to people with disabilities. However, they may also refer to other vulnerable groups at risk of social exclusion, such as young people, drug users, women, immigrants, senior citizens, ethnic minorities, prisoners, homeless people, and homosexual athletes [9,62]. Most articles addressed people/athletes with disabilities in general, but some only addressed Paralympic athletes [25,41,54], athletes with an intellectual disability [39,48], and also people with mental illness [53].

Research has shown that contact with people with disabilities may be the most important measure to promote the formation of positive attitudes towards the inclusion of people with disabilities [8,29,39,63]. Increased awareness has helped to establish accessible sports facilities and organized sport programs for people with disabilities, and this has been shown to reduce marginalization [8]. Commercial interest in sport is driven by proximity to spectators, who become consumers, and the desire of companies to target these people to sell their products. Unfortunately, the role of marketing Paralympic sport is the first perspective that fails in most countries. But the media is one of the catalysts for the commercial perspective of Paralympic sport and funding from governing bodies [41,64,65,66,67] that can achieve change.

### 4.8. Best Practices

Throughout the articles, we have come across the distinction between social inclusion in sport and through sport. Marivoet [22] defines social inclusion in sport as the actual presence of equal opportunities in accessing vulnerable people. In this regard, good practices pay attention to non-discrimination on the basis of race, ethnicity, religion, disability, gender, sexual orientation, social class, or other grounds. It further states that social inclusion through sport refers to the development of personal, social, or physical activity, or other abilities. The best practice here aims to promote formative sport, which means that the ethical principles of sport and the values associated with it are paramount. [40]. The definition attempted in this paper is further developed based on actual existing cases, but also includes developmental issues.

### 4.9. Key Barriers

In the articles consulted, two main reasons are given to explain the multiple barriers faced by people with disabilities: (1) ableism and (2) the lack of concrete policies. Chouinard [68] defines ableism as ideas, practices, institutions, and social relations that presume able-bodiedness. With ability at the heart of sport, the assumption that sport is only for healthy people reflects an ableist culture seen at peak events, such as the Olympics, Paralympics, and Commonwealth Games, that privileges people with typical abilities while labelling people with impairments as deficient and undesirable. Previous policy failures in New Zealand suggest that it is worth questioning the extent to which disability sport is characterized by ableist structures [69]. Social integration of people with disabilities in sport organizations, particularly mainstream sport clubs, is still an unacknowledged topic in sport science research and probably reflects a lack of interest from this scientific community, as it may be too much ableist-oriented [43]. Removing the distinction between disability and mainstream sport thus requires a significant rethink, as the focus is still on the normative non-disabled body [52].

The political context, with the functions, objectives, and characteristics of public policies, can also facilitate or hinder the practice of sport for people with disabilities [62], with knowledge or lack of awareness of the issue of social inclusion in sport playing an important role. In most public sport policies, countries and governments are influenced by international recommendations, so they have made the development and promotion of equality and inclusion a requirement. This is an ideal that can guide governments, policy makers, and community leaders to prevent and mitigate the marginalization of vulnerable social groups [7,9,10,11,12,13,45,46]. Legislation often assigns a leading role to sports clubs and associations in providing sports opportunities. These policies are mostly rhetorical and based on poorly developed and unclear justifications. They lack clear explanations and use vague terminology and methodology, making them confusing and open to different views and interpretations. These national policies do not explain how inclusion can be operationalized in practice [50]. Moreover, they are not specific to ability/disability and do not include specifics for underrepresented or vulnerable groups. Years ago, questions were raised about where disability sports fit into the governance structures of the United States Olympic Committee and how funds would be reallocated to support the new structure [58], but today we know that the Olympic and Paralympic Committees are in agreement (Source: https://www.usopc.org/about-the-usopc (accessed on 27 July 2023)). Creating space for interpretation results in institutional, environmental, and social barriers that limit the participation of people with disabilities in sport and increase their marginalization and discrimination in society [8,11,13,19,20,21,22,24,28,39,40,42,45,46,47]. The unchanged participation of people with disabilities in sport is defined as a serious sociopolitical situation that requires new approaches. Therefore, a more critical and theoretically grounded approach to social inclusion is needed to avoid focusing on the notion of assimilation instead of standing for equality and social justice. Unfortunately, not all associations have the human and material resources for adequate social inclusion. However, they are interested in assimilation, if they receive government funding to implement the changes. According to reports and research, few resources are available for structural improvements of facilities and coaching hours to improve social inclusion in sport [13,24,47].

Lack of awareness and professional training is a barrier to participation in physical activities by people with disabilities [12,43,51,62]. Disability is ignored in many mainstream coach educations programs, and a lack of competence is often observed among many coaches [46,47]. Inadequate funding is also cited as a major obstacle, with only modest funding available for structural improvements to facilities and for coaching hours dedicated to improving the inclusiveness of programs [12,13,19,47,51]. Policy makers need to ensure adequate financial support for high participation and inclusion expectations, adapted sports facilities, professional guidance, a focus on participation rather than competition, and the provision of more social competitions [19,45,46,47,62,70]. For sport practices to be inclusive and linked to equity, policy development needs to be participatory, with input from people with disabilities who bring their knowledge, experience, and practice, to engage with their own perspectives [45,70,71]. Milani & Starepravo [72] emphasize that regardless of the usefulness of sport for rehabilitation, income, educational purposes, or recreational activities, it is necessary that projects and programs are supported by government policies that enable social interaction between people with and without disabilities. [72]. Grandisson et al. [44] add that practicing sport to foster positive and meaningful relationships with a sense of belonging that can extend beyond the sports fields requires stakeholders from specialized, semi specialized, and regular institutions to pool their resources to develop innovative programs.

There were several limitations that are worth mentioning. Although the search included four databases, only peer-reviewed articles and not books were included in the scoping review, so some relevant literature may have been excluded. Secondly, several research members monitored the selection process for the scoping review, which may lead to inconsistencies, as different experts may suggest different topics and categories.

This scoping review should be a step towards conducting future research to narrow the acknowledged gap between policy and practice. Given the international requirements and concerns around the issue, future initiatives should focus on providing additional information on how sports clubs can be funded to include people with disabilities.

## 5. Conclusions

We do not claim that the attempt to define social inclusion in sport is definitive or complete. Rather, it is a first attempt that highlights elements of reflection that may contribute to a better definition to promote social inclusion of people with disabilities in sport, and through which we aim to encourage other researchers and decision makers to contribute to a better definition. In order to arrive at an effective definition, further studies are needed that focus on what would really contribute to change or progress.

The attempted definition is aimed at decision makers around the world, as they are the ones who can enable the conditions (social, financial, and contextual) and give clear meaning to an equal understanding of the field.

## Figures and Tables

**Table 1:** The eighteen (18) selected articles that attempt to define social inclusion in sport for peoples with disabilities.

Year, Author		Title	Selected Statements for Definition
1	2013, McConkey et al. [39]	Promoting social Inclusion Through Unified Sports for Youth with Intellectual Disabilities: a Five-Nation Study	(1) Programs like Unified Sport create bonds and promote sports values. (2) Careful matching of athletes with partners is key to success. (3) Focus on less demanding sports disciplines and on non-competitive activities.
2	2014, Marivoet [40]	Challenge of Sport towards Social Inclusion and Awareness-Raising Against any Discrimination	(1) Sports organizations as spaces of inclusion. (2) Study exclusion and discrimination in sport to gain empirical knowledge of reality. (3) The monitoring of ongoing projects of inclusive sport would result in surplus value to find validated criteria for inclusion in and through sport.
3	2015, Marques et al. [41]	The Media Approach to Paralympic Sport: Perspectives of Portuguese Athletes	(1) Solving questions between amateurism and professionalism, lawful sporting body and lawful use of the body and specific or popular sports. (2) Media is an important factor for the development possibilities of paralympic sport.
4	2016, Geidne and Jerlinder [28]	How Sports Clubs Include Children and Adolescents with Disabilities in Their Activities: A Systematic Search of Peer-Reviewed Articles	(1) Sports clubs as attractive environments for physical activity and the promotion of social and mental health. (2) Sport should be accessible to everyone, depending on their circumstances. (3) Other obstacles to the participation of disabled children and young people in organized sports can be: not all sports clubs accept disabled children and young people; managers lack adequate training; parents fear their children will be hurt or poked, supply and availability are limited.
5	2017, Haudenhuyse [42]	Introduction to the Issue “Sport for Social Inclusion: Questioning Policy, Practice and Research”	(1) Inclusion is based on exclusion: 1—inclusion is “merely” raising the level of participation of certain target/problem groups and correcting the supposed personal deficits of these groups; 2—the exclusionary mechanisms of such policies and practices remain largely unproblematized and understudied; 3—inclusion is possible as long as the “excluded” conform to the prevailing norms. Due to a lack of understanding of social inclusion, interventions in sports often perpetuate a society that creates tension. (2) Study topics: 1—the use of sport for people of “deep social exclusion”, with an emphasis on refugees and the disabled; 2—critical theory of social inclusion/exclusion in sport; 3—exploring attitudes, contexts, experiences, and assumptions regarding sport and young people at risk of social exclusion; 4—organizational and political issues related to social inclusion/exclusion in sport. (3) Sport can also be a place of exclusion within the social inclusion program. The researchers emphasize the need to question dominant assumptions that support separate sports: 1—more research is needed at the micro and macro levels; 2—the need for an intersectional theory; 3—sports coaches play an important role in establishing and maintaining a supportive environment.
6	2018, Allan et al. [25]	Narratives of Participation Among Individuals with Physical Disabilities: A life-course Analysis of Athletes’ Experiences and Development in Parasport	(1) People are fully and effectively engaged when they engage in an activity to the extent that suits them (quantity) and have a positive subjective experience (quality). (2) Six elements of participation: autonomy, belonging, challenge, commitment, mastery, and meaning. (3) Different meanings of participation lead to different ways of achieving quality in parasport, while the elements are also diverse, dynamic, and fluid over time. (4) The need to feel equal and valued.
7	2019, Albrecht et al. [43]	Sports Clubs as a Medium for Integrating People with Disabilities	(1) Participation in competitive sports is associated with higher scores in the “interaction” dimension of social integration. People with more complex needs often experience more limiting factors for participation in competitive mainstream sports. (2) Disabled people who play sports in a separate group achieve lower results compared to people who play sports together with non-disabled people. (3) For the “understanding/acceptance” and “identification” dimensions of social integration, there are no differences between the different types of sports groups.
8	2019, Grandisson et al. [44]	Strategies to Foster Inclusion Through Sports: A Scoping Review	(1) For this to be possible, we believe stakeholders from specialized, semi-specialized, and mainstream settings will need to bring their resources together to develop innovative programs. (2) Programs and policies to foster social inclusion of this population (people with ID) are essential. (3) Three key concepts inherent to the social inclusion of individuals with intellectual disability include participation in one’s community, positive interpersonal relationships, and a sense of belonging.
9	2019, Kirakosyan [45]	Sport for All and Social Inclusion of Individuals with Impairments: A Case Study from Brazil	(1) Social inclusion can be a divisive social practice. (2) Social inclusion as a fundamental principle of the UN Convention on the Rights of Persons with Disabilities. (3) Lack of consensus among scholars, practitioners, and policy makers on what constitutes social inclusion.
10	2021, Christiaens and Brittain [46]	The Complexities of Implementing Inclusion Policies for Disabled People in U.K. Non-Disabled Voluntary Community Sports Clubs	(1) Equal participation opportunities; a unique partnership approach; constant change; and inclusion outcomes: parallel inclusion, full inclusion, and choice (criticizing the social model of disability; personal experiences; inclusion is about recognizing the different needs and desires). (2) Four distinct approaches of VSC: 1—inclusion of the able-bodied, 2—removal of barriers, 3—creation of opportunities, and 4—construction of a shared identity. (3) Government and other strategic organizations often discuss social inclusion in sports using vague and broad terminology, with the implicit assumption that the reader knows what is meant. This is often problematic, as the results of the inclusion are not necessarily in line with their original intent.
11	2022, Hammond [47]	The Relationship Between Disability and Inclusion Policy and Sports Coaches’ Perceptions of Practice	(1) Definition: inclusion as a pillar of social justice, which “involves adopting a broad vision of sport for all by addressing the spectrum of needs of all, including those vulnerable to marginalization and exclusion”. (2) An inclusive coaching/sporting philosophy means that sports clubs implement programs that ensure that all athletes, regardless of ability, can reach their full potential. (3) Individual volunteers or advocates for change are crucial in establishing provision in clubs. There is a need to move away from narrow forms of participation consisting of winning and elite success.
12	2022, Hao and Razman [48]	Family Factors Associated with Physical Activity in Children with Intellectual Disability: A Systematic Review	(1) Therefore, an accessible program suitable for children with ID is the way forward, as the provision of an inclusive program and policy will promote children’s PA by allowing parents to alleviate or even remove the stressors associated with caring for children with ID. (2) Information on family factors will play a critical role in the healthy development of this vulnerable group. (3) It not only can provide valuable insights for the limited knowledge base of children with ID, but also possibly act as a reference for health professionals in relevant fields to formulate policies and generate new ideas to design tailored family intervention programs for children with ID.
13	2022, Townsend et al. [49]	Infusing Disability into Coach Education and Development: A Critical Review and Agenda for Change	(1) In Parasport, coaches are recognized at the highest level of international sport policy as performing a central role in achieving important sporting and social outcomes related to disabled people. (2) Furthermore, disability is a priority area in a number of national sports policies, moving disabled people from the margins of sport to the forefront of inclusive practices. In placing greater emphasis on expanding opportunities for participation and performance, sports organizations must encourage a clear focus on the development of a skilled and confident workforce to deliver social policy. (3) Inevitably, discussions about reforming coach education reflect deeper questions related to the ways in which disability is understood and positioned within organizational policy, sports programs, and social practice.
14	2023, Anderson et al. [50]	The Pre-Stage of Inclusion—Conditions for the Mainstreaming Process of Parasports within the Swedish Floorball Federation	(1) Defining the meaning of inclusion in the pre-stage, regarding both policy and practice, is a pressing matter. (2) How practitioners are interpreting and “conducting” sport policy decisions, meaning how they are approaching and negotiating policies of inclusion into practical implementations. (3) Enabling conditions, such as a general benignity towards inclusion, limiting conditions such as mainstream representatives lack of knowledge about the process, which can lead to further marginalization of PWD.
15	2022, Darcy et al. [51]	Disability Inclusion in Beach Precincts: Beach for all Abilities—a Community Development Approach through a Social Relational Model of Disability Lens	(1) Critics suggest that many approaches to community development through sport are instigated through a top-down delivery via broad policy frameworks that are without context or consideration of inclusion or participation of locally community-based people and their needs. (2) While policy and legislation are significant driving forces in enabling the development and integration of disability inclusion programs, organizers of existing nondisabled programs may entrench nondisabled cultural norms and resist any changes that could impact primary members of their sport organizations. (3) Asset-based community development has been adopted in community sport development as the main guiding principles and practical needs, providing a way to understand and mobilize community assets in a shared vision of beneficial social change.
16	2023, Dyer and Sandford [52]	‘Just Another Outing in a Boat’: Findings from the Evaluation of the Mixed Ability Sport Development Programme	(1) Defining the key tenets of mixed ability sport allows us to unpack what makes it different from, for example, dedicated disability sports provision, and Fitzgerald would see this model of provision as harnessing the potential of sport to challenge norms around separating disabled people in society and aligning with broader disability rights movements. (2) It was evident from the data that there was some difficulty among participants in understanding and articulating exactly what MA sport is. (3) However, many of these “welcoming” clubs assumed that they were already inclusive and were not aware of the barriers that were, often unintentionally, preventing others from joining.
17	2023, Eather et al. [53]	The Impact of Sports Participation on Mental Health and Social Outcomes in adults: a Systematic Review and the "Mental Health through Sport" Conceptual Model	(1) The identification of the mechanisms responsible for such effects (mechanisms through which social relationships and social support improve physical and psychological well-being) may direct future research in this area and help inform future policy and practice in the delivery of sport to enhance mental health and social outcomes among adult participants. (2) In summary, there is consistent evidence that sports participation is related to lower depression scores. (3) The findings of this review endorse that participation in sport is beneficial for psychological well-being, indicators of psychological ill-being, and social outcomes in adults.
18	2023, Pankowiak et al. [54]	National Paralympic Sport Policies Influencing a Country’s Paralympic Success	(1) Common national policy interventions include national government funding for sport and elite sport, effective national sport governance, grassroots sport participation, talent identification and transfer, programs for holistic development of athletes and career support, coach provision and development, and facilities. (2) This research provides evidence for the potential importance of these policies in the Paralympic domain, and suggests that a conceptual framework of Paralympic sport policy may need to assess key alignments of policy interventions in the Paralympic and Olympic domains. (3) Findings confirm that existing national Olympic sport policies are also important for Paralympic success, however, within these policies, parasport-specific processes were identified, and two policy interventions unique to Paralympic sports were found: integration of disability-specific and Paralympic sport knowledge in the sporting system, and a national framework for Paralympic athlete classification.

**Table 2:** The six categories of key statements and the 10 extracted keywords.

Key Categories (Number of Statements Found)	Selected Key Statements	10 Extracted Definition Keywords
Policy (29)	(1) Common national policy interventions include national government funding for sport and elite sport, effective national sport governance, grassroots sport participation, talent identification and transfer, programs for holistic development of athletes and career support, coach provision and development, and facilities. (2) Critics suggest that many approaches to community development through sport are instigated through a top-down delivery via broad policy frameworks that are without context or consideration of inclusion or participation of local community-based people and their needs. (3) Many project coordinators found that budgetary constraints of the partner organizations limited the success of their project, often due to a lack of available support staff and resources to build capacity.	sport, policy, disability, inclusion, organizations, exclusion, sports, policies, provision, needs
Fundamental conditions (28)	(1) Social inclusion in sport works if all actors are involved: clubs, schools, the local community, healthcare, sponsors, and others who can contribute to the development and strengthening of the community’s promotional work. (2) There are six elements of participation: autonomy, belonging, challenge, commitment, mastery, and meaning. (3) Five strategies were identified: 1—develop Unified Sports, 2—develop peer-support programs, 3—facilitate participation as an athlete in mainstream activities, 4—facilitate participation as a fan in mainstream activities, and 5—conduct activities to raise awareness.	sport, sports, inclusion, athletes, participation, disability, activities, participants, athlete, disabilities
Key elements (30)	(1) Media is an important factor. (2) The model of social inclusion through participation in sports and physical activities consists of: meaningful roles, inclusive contexts, and enabling supports. (3) The data showed that it is also important that MA (mixed ability) sport provision be regular, frequent, and sustained over time.	sport, sports, inclusion, participation, ability, disability, activities, provision, mainstream, experience
Soft skills (20)	(1) Coaches with the right skills and attitude. (2) Government and local authorities provide sufficient availability. (3) For the “understanding/acceptance” and “identification” dimensions of social integration, there are no differences between the different forms of sports groups. Without recognizing everyone as an equal individual, social inclusion can hardly progress.	sports, sport, participation, activities, individual, findings, skills, approach, athletes, inclusion
Field gaps (31)	(1) Thoughts that inclusion is an unreachable goal were expressed, where the description of inclusion as a utopia could be the result of a lack of knowledge regarding responsibilities and implementations. (2) These include “committed guardians” that maintain the exclusive nature of sport, the “prominence of a normative non-disabled body”, and the very infrastructure of sport, which promotes separation. (3) Ableist perspectives—which positively value able-bodiedness and render disability as somewhat ‘less’—have been shown to shape dominant understandings of what particular bodies are able—and not able—to do.	sport, sports, coach, inclusion, disability, coaches, coaching, need, norms, mainstream
Best practices (14)	(1) An inclusive approach to meet the diverse needs of a mixed group using the “Empowerment model”. (2) The “Mixed Ability Model” as an innovative approach to inclusive sport (disabled and non-disabled players interacting in a normal sports club environment) has great potential to achieve inclusive results. (3) In other contexts as well, MA (mixed ability) participants who identify as non-disabled perceived themselves as equal participants, who were benefiting from being involved themselves.	approach, empowerment, barriers, sport, sports, outcomes, participation, ability, participants, activities, mixed-ability, individual, disability

**Table 3:** The five categories containing 24 keywords for the definition of social inclusion in sport for people with disabilities.

	Keywords	Key People	Key Environments	Key Way to Use	Key Benefits	Key Barriers
1	ability	athletes	mainstream	ability	mixed-ability	disabilities
2	activities	coaches		activities	inclusion	exclusion
3	approach	organizations		approaches	individual	
4	athletes	participants		coaching	outcomes	
5	coaches			experiences	participation	
6	coaching			findings	provision	
7	disabilities			norms	sport	
8	exclusion			policy	needs	
9	experience			skills		
10	findings					
11	inclusion					
12	individual					
13	mainstream					
14	mixed-ability					
15	needs					
16	norms					
17	organizations					
18	outcomes					
19	participants					
20	participation					
21	policies					
22	provision					
23	skills					
24	sport					

## Data Availability

No new data were created or analyzed in this study. Data sharing is not applicable to this article.

## Supplementary Materials

The following supporting information can be downloaded at: https://www.mdpi.com/article/10.3390/healthcare11162292/s1, Table S1: The eighteen selected articles that attempt a definition of social inclusion in sports for people with disabilities; Table S2: The six categories of key statements and the 10 extracted keywords.
